# Agouti Related Peptide Secreted Via Human Mesenchymal Stem Cells Upregulates Proteasome Activity in an Alzheimer’s Disease Model

**DOI:** 10.1038/srep39340

**Published:** 2017-01-04

**Authors:** Na Kyung Lee, Sang Eon Park, Soo Jin Kwon, Sangmi Shim, Yeji Byeon, Jong-Hwa Kim, Duk L. Na, Jong Wook Chang

**Affiliations:** 1 Department of Health Sciences and Technology, SAIHST, Sungkyunkwan University, Seoul, Republic of Korea; 2 Department of Neurology, Samsung Medical Center, Sungkyunkwan University School of Medicine, Seoul, Republic of Korea; 3 Neuroscience Center, Samsung Medical Center, Seoul, Republic of Korea; 4 Stem Cell & Regenerative Medicine Institute, Samsung Medical Center, Seoul, Republic of Korea; 5 Department of Biomedical Sciences, Seoul National University, Seoul, Republic of Korea; 6 Department of Obstetrics and Gynecology, Samsung Medical Center, Seoul, Republic of Korea

## Abstract

The activity of the ubiquitin proteasome system (UPS) is downregulated in aggregation diseases such as Alzheimer’s disease (AD). In this study, we investigated the therapeutic potential of the Agouti-related peptide (AgRP), which is secreted by human mesenchymal stem cells (MSCs), in terms of its effect on the regulation of proteasome activity in AD. When SH-SY5Y human neuroblastoma cells were co-cultured with MSCs isolated from human Wharton’s Jelly (WJ-MSC), their proteasome activity was significantly upregulated. Further analysis of the conditioned media after co-culture allowed us to identify significant concentrations of a neuropeptide, called AgRP. The stereotactic delivery of either WJ-MSCs or AgRP into the hippocampi of C57BL6/J and 5XFAD mice induced a significant increase of proteasome activity and suppressed the accumulation of ubiquitin-conjugated proteins. Collectively, these findings suggest strong therapeutic potential for WJ-MSCs and AgRP to enhance proteasome activity, thereby potentially reducing abnormal protein aggregation and delaying the clinical progression of various neurodegenerative diseases.

The proteasome is a large (megadalton) and critical complex of proteolytic machinery responsible for protein turnover and cellular homeostasis in eukaryotes^1^,^2^. Through the ubiquitin proteasome system (UPS), normal and damaged proteins are selectively tagged by ubiquitin proteins, leading to their targeted degradation^3^. The 19S regulatory subunit of the 26S proteasome recognizes the polyubiquitinated chain and then unfolds the selective protein, degrading it with the 20S catalytic subunit^4^,^5^,^6^. Increasing evidence suggests that suppressing the activity of this system can lead to protein aggregation diseases such as neurodegenerative disorders^7^,^8^,^9^,^10^. Extracellular amyloid plaques can induce the generation of protein aggregates such as intracellular neurofibrillary tangles; amyloid plaques and neurofibrillary tangles are prominent hallmarks of Alzheimer’s disease (AD)^5^,^11^ which is the most common form of dementia^12^. The aggregation of abnormal proteins impairs the proteasome^13^, which can further compromise protein turnover^14^. Because of this pathological feedback loop, upregulating the proteasome system is one of the most promising ways to potentially treat neurodegenerative disorders. While proteasome inhibitors, such as bortezomib, have been well characterized, there is only limited understanding of the mechanism(s) that activates or enhances the proteasome system^15^,^16^.

Our group investigated the paracrine effects of human mesenchymal stem cells (MSCs) in terms of their proteasome regulation. Prior work has suggested potential therapeutic value of MSCs for treating various diseases including neurodegenerative disorders^17^,^18^. MSCs can influence other cells in their external surroundings by secreting paracrine factors or cytokines, causing effects ranging from anti-apoptosis, anti-inflammation, and neurogenesis activation^19^,^20^. MSCs have also shown the capability to drive the recovery and regeneration of neurological lesion sites. Previous work from our group has shown that the secretion of soluble intracellular adhesion molecular-1 (slCAM-1) by MSCs transplanted in the hippocampus of a transgenic AD mouse model causes increased expression of neprilysin, an amyloid-degrading enzyme^18^. Despite this promising finding, however, only a few studies have studied the impact of MSCs and their paracrine activities on the activity of the proteasome system and the accumulation of both intracellular and extracellular protein aggregates.

In this study, we first assessed changes in the proteasome activities of human SH-SY5Y neuroblastoma cells following co-culture with human Wharton’s Jelly-derived MSCs (WJ-MSC) and a cytokine of interest secreted by WJ-MSCs *in vitro*. Additionally, we measured the impact of separately administering both WJ-MSCs and their secreted cytokine of interest into the intra-parenchymal region in a transgenic AD mouse model (5XFAD) in order to further investigate their capacity to alter proteasome activity *in vivo*.

## Results

### Human WJ-MSCs Upregulate the Proteasome Activity of SH-SY5Y Cells

**Figure 1:**
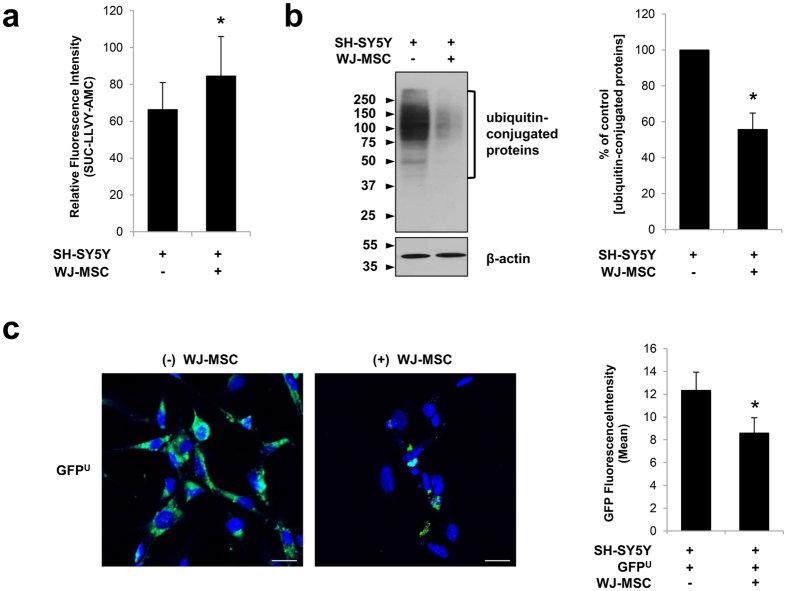
Enhancement in proteasome activity of SH-SY5Y cells following co-culture with human WJ-MSCs. **(a)** SH-SY5Y human neuroblastoma cells were co-cultured with human WJ-MSCs for 24 hrs. After sonication using retic buffer, cell lysates were treated with SUC-LLVY-AMC, and fluorescence was measured at 4 hrs. A significant increase in chymotrypsin-like activity was observed for SH-SY5Y cells co-cultured with WJ-MSCs. **(b)** Remaining cell lysates were mixed with equal volumes of urea buffer to prepare the samples for Western blotting. As depicted through densitometric analysis, compared to control SH-SY5Y cells, a decrease in ubiquitin-conjugated proteins was observed for SH-SY5Y cells co-cultured with WJ-MSCs. ^*^P < 0.05 versus SH-SY5Y alone; mean ± S.E.M.; n = 5 independent experiments. **(c)** Confocal microscope images of SH-SY5Y cells transfected with GFP^U^ (left) for 24 hrs and then co-cultured with WJ-MSCs for an additional 24 hrs (right). A reduction in fluorescence intensity (GFP) was observed after WJ-MSC co-culture. ^*^P < 0.05 versus GFP^U^-transfected SH-SY5Y; mean ± S.E.M.; n = 6 independent experiments.

To assess the capacity of human WJ-MSCs to regulate proteasome activity, SH-SY5Y human neuroblastoma cells were co-cultured with WJ-MSCs that were seeded in a transwell insert (1-μm-diameter pores). After 24 hr of incubation, we quantified changes in proteasome activity by preparing SH-SY5Y cell lysates and sequentially treating them with a fluorogenic chymotrypsin substrate, SUC-LLVY-AMC. Chymotrypsin-like activity is known as one of the predominant catalytic activities of the 20S proteasome^21^. Interestingly, compared to control SH-SY5Y cells (66.32 ± 14.68), we observed a 1.27-fold increase in proteasome activity when the cells were co-cultured with WJ-MSCs (84.55 ± 21.54) (Fig. 1a). Consistently, Western blotting showed a decrease in ubiquitin-conjugated proteins (55.71 ± 9.21%) (Fig. 1b), corroborating the upregulation of proteasome activity.

An accumulation of ubiquitin-conjugated proteins suggests significant impairment of the proteasome system^5^. The measured intensity of SH-SY5Y cells transfected with GFP^U^ and co-cultured with WJ-MSCs was 30.43% lower than that of the control cells (Fig. 1c). This reduction following WJ-MSC co-culture indicates an increase in GFP^U^ degradation, implying that WJ-MSC enhanced the proteasome activity. Together, these observations suggest that WJ-MSCs upregulate overall proteasome activity in neuronal cells, possibly by secreting soluble paracrine factors.

Along with the assessment of chymotrypsin-like activity, the potential effects of WJ-MSC co-culture on caspase, trypsin-like, and deubiquitinating activities were further examined. WJ-MSC co-culture did not have a significant effect on the caspase-like activity of SH-SY5Y cells while a slight reduction in trypsin-like activity was noted (Supplementary Figure 1a). Following WJ-MSC co-culture, deubiquitinating activity was also unaffected when observed at different reaction terminating time points (Supplementary Figure 1b).

### The Cytokine Profile is Modified Following WJ-MSC Co-Culture

**Figure 2:**
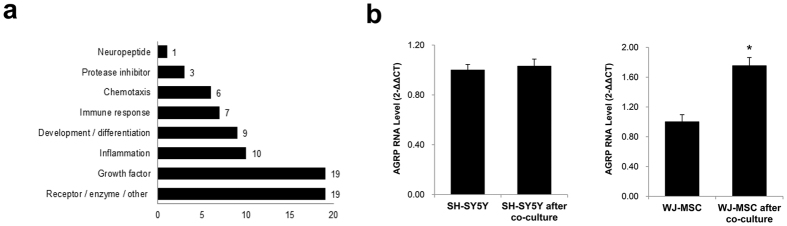
WJ-MSCs over-express the cytokine AgRP following co-culture with SH-SY5Y cells for 24 hrs. Conditioned media were collected and concentrated from these three groups: SH-SY5Y, WJ-MSC alone, SH-SY5Y and WJ-MSC after co-culture, prior to performing cytokine array (Raybiotech). (**a**) Profile of cytokines with a ≥10-fold increased secretion by WJ-MSCs following co-culture with SH-SY5Y cells. Fold increase was calculated using equation (1). (**b**) Total RNA was isolated from the harvested cells of each of the three groups using TRIzol, and quantitative reverse transcription PCR was performed to identify the origin of AGRP production and to quantitate its expression. WJ-MSCs showed a greater expression of AGRP after co-culture. ^*^P < 0.05 versus SH-SY5Y or WJ-MSC alone; mean ± S.E.M.; n = 4 independent experiments.

In order to identify the set of cytokines with higher expression levels after co-culture, we performed a cytokine array. We collected the conditioned media from the following groups to perform the array: SH-SY5Y alone, WJ-MSC alone, and SH-SY5Y cells co-cultured with WJ-MSCs. We were able to assess the media from the co-culture group because the restricted pore size of the transwell system limits cell migration across the membrane, but allows soluble proteins to be transported across the chambers. Comprehensive cytokine profiles were obtained from each of the groups, the values were normalized against a positive control, and the protein sample contents were corrected for background. WJ-MSC co-culture-induced change in cytokine expression was calculated respective to the WJ-MSC alone group by using equation (1) and was expressed as fold-change compared to that group. Of the various cytokines identified, the Agouti-related peptide, AgRP, was a neuropeptide detected in the conditioned media of SH-SY5Y cells co-cultured with WJ-MSCs (Fig. 2a). AgRP is produced by NPY/AgRP neurons in the brain and is widely known for its role in stimulating food intake^22^. Its connection to the UPS, however, has not yet been reported.

To further confirm the secretion of AgRP by WJ-MSCs, we isolated total RNA from each of the cell types after co-culture, and the expression of AGRP was analyzed by quantitative reverse transcription polymerase chain reaction (qRT-PCR). AGRP expression did not show a dramatic difference between the control cells (1.00 ± 0.044) and SH-SY5Y co-cultured with WJ-MSCs (1.03 ± 0.057) (Fig. 2b). WJ-MSCs, by contrast, expressed AGRP in minimal levels when cultured alone (1.00 ± 0.096), but showed significantly higher expression (1.75-fold increase) following co-culture with SH-SY5Y cells (1.75 ± 0.11). These results demonstrate that co-culture with SH-SY5Y neuroblastoma cells boosts the expression and secretion of AgRP by WJ-MSCs.

### AgRP Enhances Proteasome Activity

**Figure 3:**
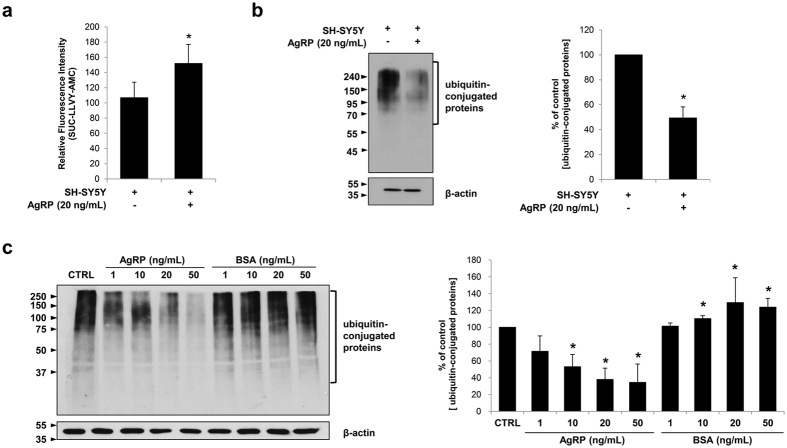
AgRP treatment upregulates the proteasome activities of SH-SY5Y cells *in vitro*. (**a**) SH-SY5Y neuroblastoma were treated with the recombinant human protein AgRP (20 ng/mL) for 24 hrs in a serum free state. ^*^P < 0.05 versus SH-SY5Y only; mean ± S.E.M.; n = 4 independent experiments. Harvested cells were sonicated using retic buffer. Cell lysates were treated with SUC-LLVY-AMC and fluorescence was measured at 4 hrs. Chymotrypsin-like activity was increased after AgRP treatment. **(b)** Remaining cell lysates were mixed with equal volumes of urea buffer to prepare the samples for Western blotting. On Western blotting, AgRP-treated cells showed a reduced level of ubiquitin conjugate proteins. ^*^P < 0.05 versus SH-SY5Y only; mean ± S.E.M.; n = 4 independent experiments. **(c**) A dose-dependent decrease in ubiquitin-conjugated proteins was observed in SH-SY5Y cells treated with varying doses of AgRP (1, 10, 20, and 50 ng/mL) for 24 hrs. The ubiquitin-conjugated proteins of the control and PBS (containing 0.1% BSA)-treated samples were not significantly different. ^*^P < 0.05; mean ± S.E.M.; n = 3 independent experiments.

We further examined the mechanism by which AgRP causes the upregulation of proteasome activity by treating SH-SY5Y cells with the recombinant human AgRP protein for 24 hrs. Compared to control SH-SY5Y cells (106.87 ± 20.40), the proteasome activity of AgRP-treated SH-SY5Y cells was enhanced (151.92 ± 25.08) by 1.42-fold (Fig. 3a). Deubiquitinating activity was slightly reduced after AgRP treatment but the effects were not dramatic (Supplementary Figure 2). SH-SY5Y cells treated with AgRP (20 ng/mL) also showed significantly lower amounts of ubiquitin-conjugated proteins (49.33 ± 8.89%) (Fig. 3b). This reduction of ubiquitin-conjugated proteins showed a dose-dependent relationship in SH-SY5Y cells treated with varying doses of AgRP (Fig. 3c). Based on densitometric analysis, ubiquitin-conjugated protein concentrations were 71.53 ± 18.34%, 53.12 ± 14.66%, 38.05 ± 13.45%, and 34.60 ± 21.85% for SH-SY5Y cells treated with 1, 10, 20, and 50 ng/mL of AgRP, respectively (Fig. 3c). As expected, SH-SY5Y cells treated with varying concentrations (1, 10, 20, and 50 ng/mL) of PBS containing BSA displayed similar or increased aggregation of ubiquitin-conjugated proteins (Fig. 3c). The differences between cells treated with AgRP and the equivalent concentration of PBS containing BSA were also statistically significant except for 1 ng/mL.

**Figure 4:**
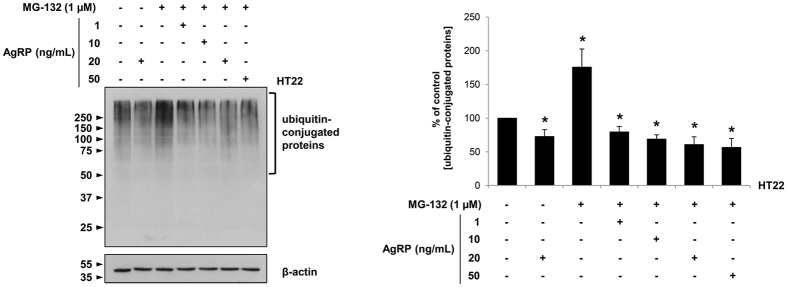
AgRP rescues proteasome activity in HT22 cells treated with MG-132. HT22 mouse hippocampal neuronal cells were co-treated with MG-132 (1 μM) and varying doses of AgRP (1, 10, 20, and 50 ng/mL) for 24 hrs in a serum-free environment. Harvested cells were sonicated in urea buffer prior to performing Western blot. While a high aggregation of ubiquitin conjugate proteins was observed in HT22 cells treated solely with MG-132, ubiquitin conjugate protein levels were rescued by increasing AgRP dose, as assessed through densitometric analysis. ^*^P < 0.05 versus HT22 only; mean ± S.E.M.; n = 4 independent experiments.

AgRP’s effect on proteasome activity was further examined by co-treating HT22 mouse hippocampal neuronal cells with the potent proteasome inhibitor MG-132^23^ and with varying doses of AgRP (1, 10, 20, 50 ng/mL). HT22 neuronal cells that received a combined treatment of MG-132 (1 μM) and AgRP showed a dose-dependent decrease in ubiquitin protein conjugates ubiquitin-conjugated proteins (Fig. 4). The ubiquitin conjugate ubiquitin-conjugated proteins concentrations were 79.46 ± 8.37%, 68.90 ± 6.32%, 60.76 ± 11.63%, and 56.60 ± 13.33% when treated with MG-132 and 1, 10, 20, or 50 ng/mL of AgRP, respectively (Fig. 4). Compared to the control, when treated with MG-132 only, the accumulation of ubiquitin-conjugated proteins increased strikingly (1.76 fold). Collectively, these data indicate a significant role of AgRP in driving the upregulation of proteasome activity for both HT22 and SH-SY5Y cells, with or without the presence of MG-132.

### WJ-MSC/AgRP-Injected Hippocampal Lysates Show Upregulated Proteasome Activity

**Figure 5:**
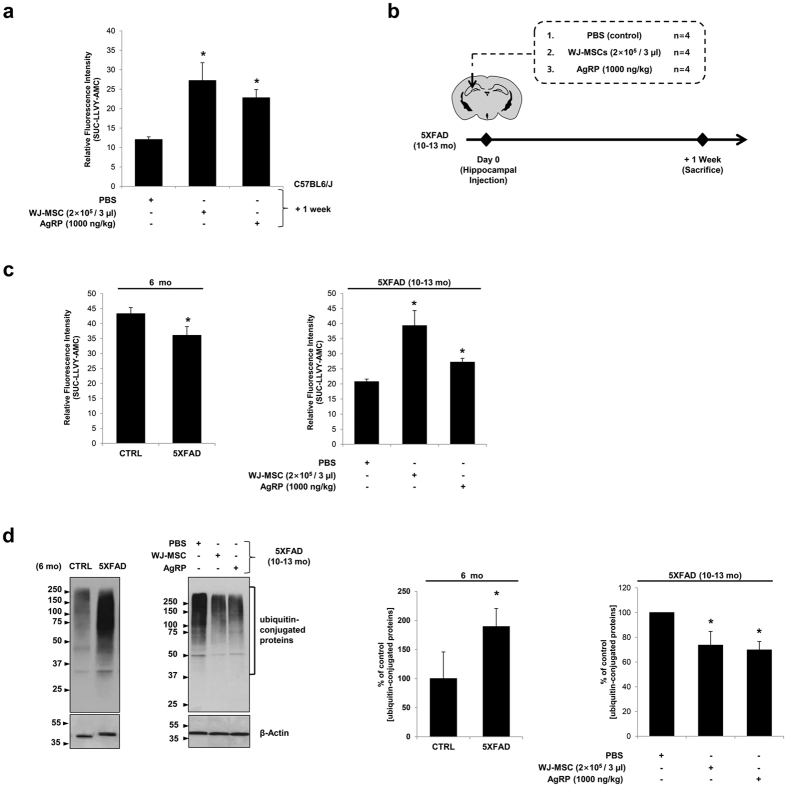
Hippocampal injections of WJ-MSCs or AgRP into 5XFAD mice enhance proteasome activity. **(a)** PBS, WJ-MSCs (2 × 10^5^/3 μl), or AgRP (1000 ng/kg) was injected into the left hippocampus of C57BL6/J mice. Mice were sacrificed one week post injection, and the hippocampal lysates were collected and treated with SUC-LLVY-AMC. Fluorescence was measured after 4 hrs of treatment. Compared to the control group, chymotrypsin activities were significantly higher in WJ-MSC and AgRP (1000 ng/kg) groups that were sacrificed at one week post-transplantation. ^*^P < 0.05; mean ± S.E.M.; n = 3 per group. (**b**) Previous results using C57BL6/J mice were used to create the study design for 5XFAD mice (10–13 mo). (**c**) Compared to age-matched controls (CTRL; 6 months), the brain lysates of 5XFAD showed a reduction in proteasome activity. Chymotrypsin-like activity was upregulated in the hippocampal tissues of 5XFAD mice (10–13 mo) injected with WJ-MSCs or AgRP. ^*^P < 0.05; mean ± S.E.M.; n = 4 per group. **(d)** Remaining hippocampal lysates were mixed with equal volumes of urea buffer to prepare the samples for Western blotting. A higher accumulation of ubiquitin conjugate proteins was observed from 6-mo-old 5XFAD mice compared to age-matched controls (CTRL). The opposite was observed in 5XFAD mice (10–13 mo) injected with WJ-MSCs or AgRP. ^*^P < 0.05; mean ± S.E.M.; n = 4 per group.

We assessed the therapeutic applications of WJ-MSCs and AgRP in neurodegenerative diseases by delivering either WJ-MSCs or AgRP directly into the left hippocampus of 10 to 13-month(mo)-old 5XFAD transgenic AD mice. Prior to this experiment using transgenic AD mice, we performed a preliminary study using C57BL6/J mice to determine the optimal AgRP dose and sacrifice time point. Compared to the PBS-injected group (n = 3), the proteasome activity of the hippocampal lysates of the WJ-MSC transplanted group (n = 3) that was sacrificed one week post injection was enhanced by approximately 2.26-fold (Fig. 5a). There was not a significant difference between the control and WJ-MSC-injected groups sacrificed one day after injection (data not shown). The AgRP-injected group sacrificed one week after injection also showed a 1.89-fold increase in proteasome activity compared to that of the control. When comparing the two AgRP doses, we found that 500 ng/kg (data not shown) was significantly less effective than 1000 ng/kg in upregulating overall proteasome activity (Fig. 5a). We thus chose a sacrifice time point of one week after injection and an injection dose of 1000 ng/kg for AgRP for all hippocampal injections performed on the following 5XFAD groups: PBS (n = 4), WJ-MSCs (n = 4), and AgRP (n = 4) (Fig. 5b).

We also assessed the proteasome activity of six-month-old 5XFAD (n = 3) and control littermate (n = 3) brain lysates in order to determine whether our observations were consistent with previous studies. We observed significant downregulation of proteasome activity and accumulation of ubiquitin-conjugated proteins in the brain lysates of 5XFAD mice. The proteasome activity of six-month-old 5XFAD mice was reduced by 19.65% compared to that of control littermates (Fig. 5c). Additionally, the amount of ubiquitin-conjugated proteins increased by 1.90-fold in 5XFAD mice (Fig. 5d) compared to control littermates. Such results are consistent with previous studies^24^,^25^.

One week following injection of PBS, WJ-MSC, or AgRP, hippocampal protein lysates of the 5XFAD groups were collected, and the proteasome activity was measured. The proteasome activity of WJ-MSC- and AgRP-injected groups was significantly upregulated by 1.90- and 1.31-fold, respectively, compared to the PBS-injected group (Fig. 5c). Moreover, compared to the control group, the concentration of ubiquitin-conjugated proteins of WJ-MSC- and AgRP-injected groups was reduced to 26.31% and 30.18%, respectively, further supporting their roles in the upregulation of proteasome activity (Fig. 5d). These *in vivo* observations reflect the extended roles of WJ-MSCs and AgRP in elevating proteasome activity not only in normal organisms, but also in disease models characterized by their impairment in proteasome activity.

## Discussion

We have shown for the first time that human Wharton’s Jelly-derived mesenchymal stem cells (WJ-MSCs) are capable of enhancing proteasome function in nearby cells by secreting a neuropeptide called Agouti-related peptide (AgRP). AgRP is commonly known to be secreted by the neuropeptide Y (NPY) neurons in the arcuate nucleus of the hypothalamus^26^,^27^. It has been studied predominantly for its role in stimulating appetite and food intake^28^,^29^; however, its association with neurodegenerative disorders remains broadly uncharacterized. Moreover, we are the first to propose the potential therapeutic capacity of AgRP or WJ-MSCs by modulating proteasome function.

Our identification of treatments that can enhance proteasome function bears great therapeutic significance. The proteasome is known to be impaired in various neurodegenerative diseases such as AD^6^,^30^; therefore, the identification of mechanisms that positively regulate proteasome activity has been a key goal of therapeutic research^31^. The upregulation of the proteasome can potentially increase the degradation of presenilin-1,-2, and any mutated forms, as well as β-secretase, all of which are critical drivers of Aβ production^32^,^33^. Through our previous works, we have demonstrated multiple times the therapeutic benefits following MSC transplantation into the hippocampus of an AD transgenic mouse, such as restoration of spatial memory assessed through the Morris Water Maze, and reduction of amyloid plaques which we hypothesized could be partially due to increased neprilysin, an Aβ degrading enzyme, or to decreased β-secretase-1 (BACE-1)^18^,^34^,^35^.

Concurrent with these findings, here we report on an alternative mechanism underlying this therapeutic benefit which is the upregulation of proteasome activity by AgRP. To directly assess the therapeutic benefits of AgRP and WJ-MSCs without having to overcome the obstacle of the blood brain barrier and dilution effects of the cerebrospinal fluid (CSF) flow, parenchymal was chosen over alternative administration routes such as the intracerebroventricular (ICV) route. It can be inferred from this study that the paracrine factors secreted by transplanted MSCs can stimulate the impaired proteasome function to prevent or slow amyloid production and decrease the amount of aggregated proteins. This indicates that transplanted MSCs can simultaneously treat both early and later stages of AD pathogenesis. Following the *in vitro* screening and validation of MSC and AgRP’s roles, the continued observation of their abilities to upregulate proteasome activity *in vivo* in a transgenic AD mouse model reinforce the significant and potential therapeutic benefits that may arise through the applications of MSCs and AgRP in AD therapy.

There are many synthetic regulators of proteasome activity that are currently available, mostly made up of proteasome inhibitors such as MG-132, epoxomicin, lactacystin, and bortezomib^21^. MG-132 and lactacystin have both been shown to increase the production of amyloid-β42 proteins^36^. Therefore, the finding that AgRP can rescue the effect of MG-132 treatment on HT22 cells is meaningful because it bolsters its therapeutic potential for treating AD. Many proteasome inhibitors have been shown to exert a profound effect on chymotrypsin-like activity^21^, which is the major activity investigated in this study. Although they have not been as widely studied as inhibitors, several proteasome activators have been investigated^37^. Oleuropein and betulinic acid have been studied; however, only betulinic acid has been reported to selectively activate the proteasome’s chymotrypsin-like activity^37^,^38^. To the best of our knowledge, this is the first study to show the upregulation of chymotrypsin-like activity by a protein such as AgRP.

Taken together, our findings indicate that human WJ-MSCs upregulate proteasome activity through their paracrine effects, specifically by secreting a neuropeptide called AgRP. These results are particularly noteworthy because the upregulation of proteasome activity induced by WJ-MSCs or AgRP led to an overall reduction of ubiquitin-conjugated proteins. This can potentially alleviate the pathological burden of diseases associated with protein aggregation, and it highlights the therapeutic potential of WJ-MSCs and AgRP. Further research is warranted to expedite the exploration of potential therapeutic applications of WJ-MSCs and AgRP as novel and promising proteasome enhancers for a wide range of diseases characterized by impaired proteasome activity.

## Materials and Methods

### Ethical Statement

This study was approved by the Institutional Animal Care and Use Committee (IACUC) of Samsung Biomedical Research Institute (SBRI) at Samsung Medical Center (SMC). As an accredited facility of the Association for Assessment and Accreditation of Laboratory Animal Care International (AAALAC International), SBRI also abides by the Institute of Laboratory Animal Resources (ILAR) guidelines. In accordance with the guidelines approved by the Institutional Review Board (IRB) of Samsung Medical Center, umbilical cords were collected with informed consent from pregnant mothers (IRB# 2015-09-023-003).

### Isolation and Culture of MSCs from Human Wharton’s Jelly

Human mesenchymal stem cells (MSCs) were isolated from Wharton’s Jelly based on previously reported procedures^39^. Under aseptic conditions, umbilical cords (kindly provided by Professor Jong -Hwa Kim) were fully washed with Dulbecco’s Phosphate Buffered Saline (DPBS; Biowest, USA) and were then cut into pieces 3–4 cm in length. After stripping the adjacent blood vessels and amnion, the tissue was minced and incubated with 2 mg/ml of collagenase (Gibco, USA) for 60–90 min and further digested in 0.25% trypsin (Gibco, USA) for 30 min at 37 °C under gentle agitation. After adding fetal bovine serum (FBS; Biowest, USA) the digested mixture was centrifuged for 10 min at 1000 g at room temperature (RT). After washing several times with serum-free Dulbecco Modified Eagle Medium (DMEM; Biowest, USA), isolated MSCs were cultured and expanded in DMEM media supplemented with 20% FBS and 1% penicillin-streptomycin (Gibco, USA) at 37 °C with 5% CO_2._ Passage 5–6 WJ-MSCs were used for the study.

### SH-SY5Y Co-culture with WJ-MSCs

SH-SY5Y human neuroblastoma cells (ATCC, USA) were grown in complete Minimal Essential Medium (MEMα1x; Gibco-Invitrogen, USA) supplemented with 10% FBS (Biowest, USA) and 0.5% gentamicin (Life Technologies, USA). At ~90% confluency, SH-SY5Y cells (lower chamber of the Transwell unit) were co-cultured with Wharton’s Jelly-derived mesenchymal stem cells (WJ-MSCs) for 24 hrs in a serum-free state at 37 °C with 5% CO_2_. WJ-MSCs were seeded (1 × 10^5^/1 mL) into the upper chamber of 6-well transwell inserts (BD Falcon, USA). After a 24-hr incubation period, cells were harvested through trypsinization (0.25%, Gibco-Invitrogen, USA) and were washed with DPBS (Biowest, USA).

### AgRP and MG-132 Treatment

SH-SY5Y cells were treated with varying doses (0–50 ng/mL) of recombinant human Agouti-related protein (AgRP; R&D, USA) in a serum-free state in 37 °C with 5% CO_2_. AgRP was reconstituted in PBS containing 0.1% bovine serum albumin (BSA), as per the manufacturer’s recommendation (R&D, USA). After 24 hrs, cells were harvested to analyze proteasome activity. To further examine the effects of AgRP on proteasome activity in alternative cell lines, we also cultured HT22, a mouse hippocampal neuronal cell line kindly provided by Professor Nam-In Baek (Kyung-Hee University, Republic of Korea). HT22 cells were cultured according to previously published procedures^40^ using DMEM (Biowest, USA) supplemented with 10% FBS, 5 mM L-glutamine, and 1% penicillin-streptomycin (Life Technologies, USA). At 90% confluency, HT22 cells were co-treated with 0.25 μM of N-benzoyloxycarbonyl (Z)-Leu-Leu-leucinal (MG-132; Calbiochem, USA), a potent proteasome inhibitor, and varying doses of AgRP (0–50 ng/mL) in a serum-free state for 24 hrs.

### GFP Degron Transfection

SH-SY5Y cells were grown up to 90% confluency in a 6-well plate and then transfected with 4.0 μg of GFP Degron (GFP^U^), which was kindly provided by Dr. Sangmi Shim (Seoul National University, Republic of Korea), using Lipofectamine 2000 (Life Technologies, USA). Cells were transfected for 24 hrs and then co-cultured with WJ-MSCs for an additional 24 hrs in a serum-free state. Images were acquired using a confocal microscope (LSM 700; Carl Zeiss AG, Jena, Germany), and fluorescence intensities were measured using the Image-J software (NIH, USA).

### RNA Isolation and Quantitative Reverse Transcription PCR

Total RNA was isolated using TRIzol (Life Technologies, USA) as recommended by the manufacturer. SuperScriptTM II Reverse Transcriptase (Invitrogen, USA) was used to convert 2 μg of RNA to cDNA. Levels of the AGRP mRNA gene transcripts were quantified (Agouti-related peptide, NM 001138) along with those of GAPDH (glyceraldehyde 3-phosphate dehydrogenase, NM 002046), which was used as an internal control. The following primers were designed and used: AGRP Forward (FOR): AGT CAC GTG TGG CCC TTC AT, AGRP Reverse (REV): TCC GGG ATT CTT GCC TAG AG, GAPDH FOR: CGA GAT CCC TCC AAA ATC AA, and GAPDH REV: CCT TCT CCA TGG TGG TGA A. Quantitative reverse transcription PCR (RT-qPCR) was performed on a Step ONE Plus system (AB, USA) using 2X Power SYBR Green Master Mix (AB, USA) under the following three stage program parameters: 95 °C 10 min, 95 °C 15 sec, 59 °C 30 sec (40 cycles). Relative gene expression was calculated using the 2^−ΔΔCT^ method proposed by Livak and Schmittgen^41^.

### Hippocampal Injections of WJ-MSCs and AgRP into 5XFAD Mice

5XFAD mice (B6SJL-Tg(APPSwFlLon, PSEN1*M146L*L286V)6799Vas/Mmjax) were purchased from Jackson Laboratories and bred and genotyped through DNA extraction from tails. Using a stereotactic apparatus (Harvard Apparatus, USA), WJ-MSCs (2 × 10^5^/3 μl suspended in MEM alpha 1x phenol red free media), AgRP (1000 ng/kg), or PBS 1x (vehicle control) were injected into the left hippocampus of both 10–13-month-old C57BL6/J (total: n = 9) and 5XFAD (total: n = 12) mice at the following coordinates: A/P −2.3 mm, M/L −1.3 mm, and D/V −2.0 mm. Injections were performed using a 25 μl Hamilton syringe at approximately 0.5 μl/min. Mice were sacrificed through cardiac perfusion one week post injection. Brains were extracted, and the left hippocampi were dissected. The hippocampal tissues were then homogenized in retic buffer to measure proteasome activity.

### 26S Proteasome Activity Assay

The proteasome assay was performed according to previously reported protocols^42^. In the absence of proteasome inhibitors, cell lysates were prepared through ultra-sonication in retic buffer (30 mM Tris (pH 7.8), 5 mM MgCl_2,_ 5 mM KCl, 0.5 mM DTT, 2 mM ATP). In 96-well black/clear plates (BD Falcon, USA), 5 μg of the cytoplasmic proteins were treated with 200 μM of the fluorogenic substrate Suc-Leu-Leu-Val-Tyr-AMC (SUC-LLVY-AMC; Enzo, Republic of Korea) to a final concentration of 100 μM. Cytoplasmic proteins were treated with the fluorogenic substrate and incubated for up to 4 hrs at 37 °C. After the incubation period, the reaction was stopped with the addition of cold ethanol, and the fluorescence of the samples was measured at 380/460 nm (Ex/Em) using a fluorometer (Glomax, Promega, USA). The following fluorogenic substrates were obtained from Enzo and were also used in this study: Bz-Val-Gly-Arg-AMC (Bz-VGR-AMC; trypsin-like activity), Ac-Gly-Pro-Leu-Asp-AMC (Ac-GPLD-AMC; caspase-like activity), and ubiquitin-AMC (deubiquitinating activity).

### Western Blot Analysis

Cell lysates were prepared by sonication with retic buffer followed by further sonication with the addition of urea buffer (7M urea, 2.8M thiourea, 4% CHAPS, 130 mM dithiothreitol, and 40mM Tris-HCl (pH 8.8)) at a 1:1 ratio. After centrifugation, the supernatant was collected, and protein quantification was performed using the Bradford assay (Bio-rad, USA). Equivalent amounts of proteins (10 μg per lane) were loaded and subjected to sodium dodecyl sulfate-polyacrylamide gel electrophoresis (SDS-PAGE). Afterward, proteins were transferred to a nitrocellulose membrane. The membrane was blocked for 1 hr using 5% skim milk (BD Difco, USA) in Tris-buffered saline with 0.1% Tween 20(TBST) and then incubated with the anti-ubiquitin antibody (Ub, 1:1000; Santa Cruz, USA) at 4 °C overnight. Anti-beta(β)-actin antibody (1:5000; Santa Cruz, USA) was used as a loading control. Afterward, the membrane was incubated with the secondary antibody (goat anti-mouse IgG-HRP; Ab Frontier, USA) for 1 hr at RT. Blots were developed using ECL (Advansta, USA), and protein bands were detected through exposure to X-ray film. Densitometric analysis was performed using Image-J software (NIH, USA).

### Cytokine Array

To assess a wide range of secretion levels for several different cytokines after 24 hrs of co-culture, conditioned serum-free media was collected and concentrated using a centricon (Millipore, USA). Concentrated media (SH-SY5Y or WJ-MSC alone and SH-SY5Y cells co-cultured with WJ-MSCs) was then analyzed using the RayBio® Human Cytokine Antibody Arrays – Biotin-Label Based G Series (Raybiotech, USA) in order to detect 509 cytokines, as per manufacturer’s instructions. The following equation was used to calculate WJ-MSC co-culture-induced expressions of cytokines (fold change):


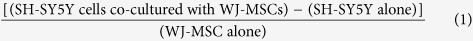


### Statistical Analysis

All data are presented as mean ± standard error of the mean (S.E.M.) A P-value ≤ 0.05 was considered to be statistically significant. Differences between groups were examined using Student’s t test.

## Additional Information

**How to cite this article**: Lee, N. K. *et al*. Agouti Related Peptide Secreted Via Human Mesenchymal Stem Cells Upregulates Proteasome Activity in an Alzheimer’s Disease Model. *Sci. Rep.* **7**, 39340; doi: 10.1038/srep39340 (2017).

**Publisher's note:** Springer Nature remains neutral with regard to jurisdictional claims in published maps and institutional affiliations.

## Supplementary Material

Supplementary Information
