# Supplementary material for: Agouti Related Peptide Secreted Via Human Mesenchymal Stem Cells Upregulates Proteasome Activity in an Alzheimer’s Disease Model
Source: Sci Rep. 2017 Jan 4;7:39340. doi: 10.1038/srep39340 (PMC5209736; doi:10.1038/srep39340)
Supplement: Supplementary Information [file srep39340-s1.pdf]

Supplementary Information

Agouti Related Peptide Secreted via Human Mesenchymal Stem Cells Upregulates Proteasome Activity in an Alzheimer's Disease Model

Na Kyung Lee[1,2,3,4], Sang Eon Park[4], Soo Jin Kwon[4], Sangmi Shim[5], Yeji Byeon[4]
Jong-Hwa Kim[6], Duk L. Na[1,2,3,4*], and Jong Wook Chang[1,4*]

[1] *Department of Health Sciences and Technology, SAIHST, Sungkyunkwan University, Seoul, Republic of Korea*

[2] *Department of Neurology, Samsung Medical Center, Sungkyunkwan University School of Medicine, Seoul, Republic of Korea*

[3] *Neuroscience Center, Samsung Medical Center, Seoul, Republic of Korea*

[4] *Stem Cell & Regenerative Medicine Institute, Samsung Medical Center, Seoul, Republic of Korea*

[5] *Department of Biomedical Sciences, Seoul National University, Seoul, Republic of Korea*

[6] *Department of Obstetrics and Gynecology, Samsung Medical Center, Seoul, Republic of Korea*

Running Title: AgRP and WJ-MSCs as Proteasome Enhancers

[*] **Address correspondence (s) to**:

(1) Jong Wook Chang, Ph.D, Stem Cell & Regenerative Medicine Institute, Samsung Medical Center, Department of Health Sciences and Technology, SAIHST, Sungkyunkwan University, 81 Irwon-dong, Gangnam-gu, Seoul 06351, Republic of Korea. Phone: +82-2-3410-3687. Email: jongwook.chang@samsung.com

(2) Duk L. Na, M.D., Department of Neurology, Samsung Medical Center, Sungkyunkwan University School of Medicine, 81 Irwon-dong, Gangnam-gu, Seoul 06351, Republic of Korea. Phone: +82-2-3410-3591, Fax: +82-2-3410-0052. E-mail: dukna@naver.com

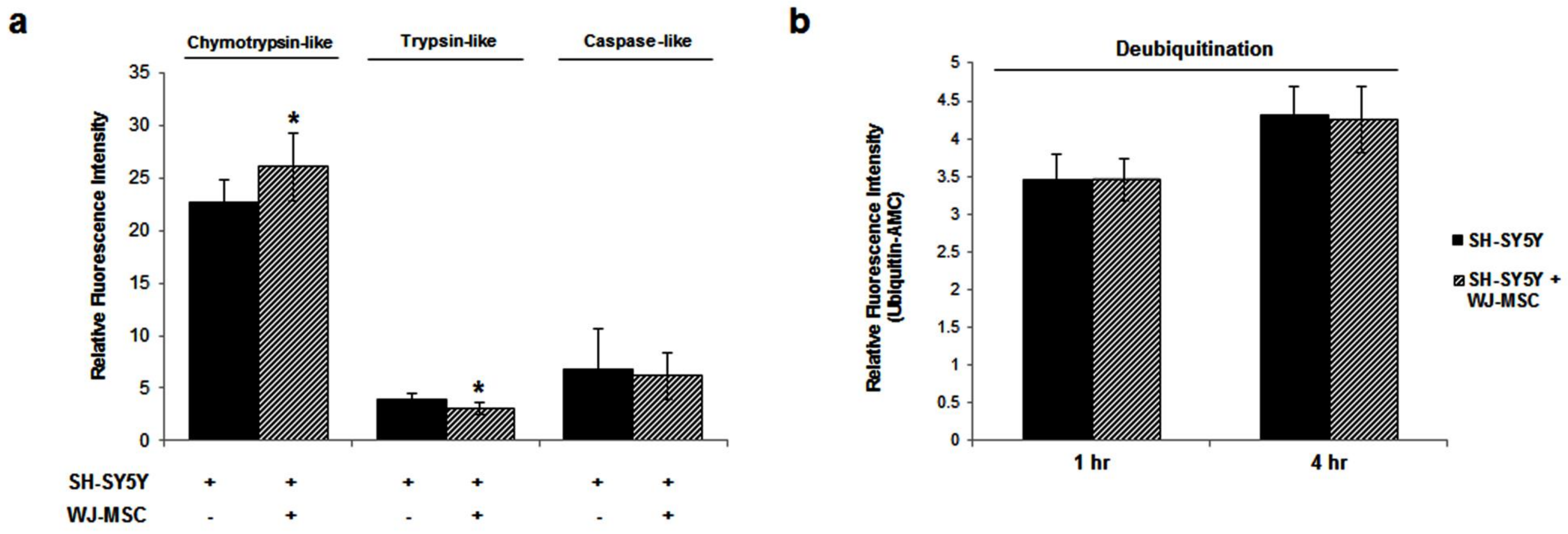

Supplementary Figure 1. Assessment on the effects of WJ-MSC co-culture on alternative proteoyltic activities. (a) SH-SY5Y human neuroblastoma cells were co-cultured with human WJ-MSCs for 24 hrs. Harvested cells were sonicated in retic buffer and the cell lysates were treated with SUC-LLVY-AMC (chymotrypsin-like), Bz-VGR-AMC (trypsin-like), or Ac-GPLD-AMC (caspase-like). Fluorescence was measured at 1 hr. $^{*}P<0.05$ versus SH-SY5Y only; mean ± S.E.M; n=3 independent experiments. **(b)** SH-SY5Y human neuroblastoma cells were co-cultured with human WJ-MSCs for 24 hrs. Harvested cells were sonicated in retic buffer and the cell lysates were treated with ubiquitin-AMC (deubiquitinating activity). Fluorescence was measured at 1 and 4 hrs. (mean ± S.E.M; n=6 independent experiments).

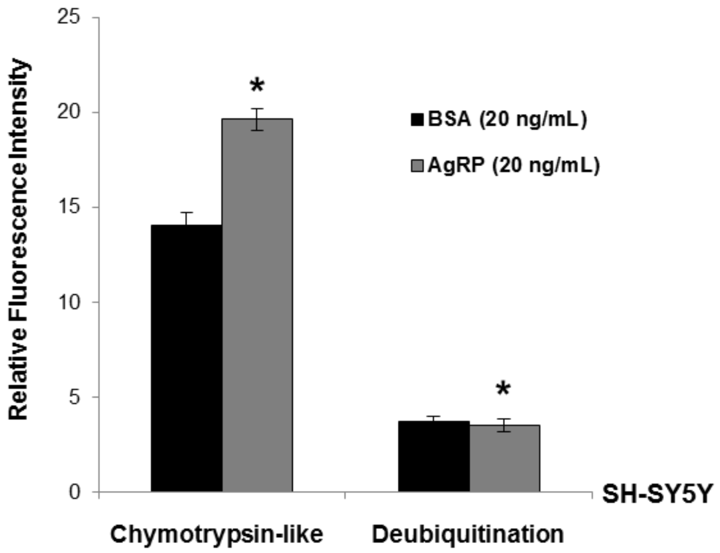

Supplementary Figure 2. Effects of AgRP treatment on deubiquitinating activity. SH-SY5Y neuroblastoma cells were treated with the recombinant human AgRP (20 ng/mL) protein or BSA (20 ng/mL) as a control for 24 hrs. Harvested cells were sonicated in retic buffer and the cell lysates were treated with SUC-LLVY-AMC (chymotrypsin-like) or ubiquitin-AMC (deubiquitinating activity). Fluorescence was measured at 4 hrs. $^{*}P<0.05$ versus SH-SY5Y cells treated with BSA; mean ± S.E.M; n=3 independent experiments.
